# Causes of death and survival analysis for patients with retinoblastoma in Jordan

**DOI:** 10.3389/fmed.2023.1244308

**Published:** 2023-09-04

**Authors:** Tharwa Bilbeisi, Razaq Almasry, Mariam Obeidat, Mona Mohammad, Imad Jaradat, Hadeel Halalsheh, Ayat Alni’mat, Danah Kanj Ahmad, Nour Alsaket, Mustafa Mehyar, Ibrahim Al-Nawaiseh, Yacoub A. Yousef

**Affiliations:** ^1^FedEx Institute of Technology, University of Memphis, Memphis, TN, United States; ^2^St. Jude Children’s Research Hospital, Memphis, TN, United States; ^3^Department of Surgery (Ophthalmology), King Hussein Cancer Center (KHCC), Amman, Jordan; ^4^Department of Radiation Oncology, King Hussein Cancer Center (KHCC), Amman, Jordan; ^5^Department of Pediatrics Oncology, King Hussein Cancer Center (KHCC), Amman, Jordan

**Keywords:** death, Jordan, metastasis, retinoblastoma, survival

## Abstract

**Purpose:**

To analyze causes and prognostic factors for death among Retinoblastoma (Rb) patients treated at a single specialized tertiary cancer center in Jordan.

**Methods:**

We reviewed the mortality causes for all Rb patients who have been treated at the King Hussein Cancer Center between 2003 and 2019 and were followed for at least 3 years after diagnosis. The main outcome measures included demographics, laterality, tumor stage, treatment modalities, metastasis, survival, and causes of death.

**Results:**

Twenty-four (5%) of the 478 patients died from retinoblastoma and 5-year survival was 94%. The mean age at diagnosis was 15 months (median, 18 months; range, 4–38 months); eight (33%) received diagnoses within the first year of life. Eleven (46%) were boys, 16 (67%) had bilateral disease, and 3 (13%) had a positive family history. The stage for the worst eye was C for 1 (4%) patient, D in 6 (25%) patients, and E (T3) in 15 (63%) patients. Two patients had extraocular Rb at diagnosis, and four of the patients who had intraocular Rb at diagnosis refused treatment and then came back with extraocular Rb. In total, extraocular disease was encountered in six eyes (six patients). After a 120-month median follow-up period, 24 patients (5%) died of second neoplasms (*n* = 3) or metastases (*n* = 21). Significant predictive factors for metastasis and death included advanced IIRC tumor stage (*p* < 0.0001), the presence of high-risk pathological features in the enucleated eyes (*p* = 0.013), parental refusal of the recommended primary treatment plan (*p* < 0.0001), and extraocular extension (*p* < 0.0001).

**Conclusion:**

The 5-year survival rates of Rb patients in Jordan are as high as those in high-income countries. However, 5% are still dying from metastatic disease, prompting the need for awareness campaigns to educate the public about the high cure rates and to prevent treatment abandonment.

## Introduction

Retinoblastoma (Rb) is the most common intraocular malignancy in children worldwide (1). It is the most common intraocular malignancy across all ages in Jordan, shown to be more common than uveal melanoma (2). Rb is a malignant tumor of the developing retina that develops from cells that have cancer-predisposing variants in both copies of *RB1*. It may be unifocal or multifocal and 40% of affected individuals have bilateral disease. Timely diagnosis and prompt management are critical for cure. The goal of Rb treatment is curing the patient of disease; however, preserving the globe and vision are secondary aims when safe and possible to do so. Rb is a complicated disease treated via systemic chemotherapy, intra-arterial chemotherapy, intra-vitreal chemotherapy, focal consolidation therapy, and other modalities (3–10).

Globally, the incidence of Rb is 1 in 15,000–20,000 live births, although variable rates have been reported in 3.4–42 cases per million (2, 11, 12). Disease-specific mortality has improved over the last 10 years (13); however, global disparities in regional mortality rates remain. In an analysis of the gross national income of the country versus Rb mortality, Chantada et al. reported that survival from Rb is 30% in low-income countries, 60% in lower-middle-income countries, 75% in upper-middle-income countries, and 95% in high-income countries (14). Recently, Gündüz et al. reported an overall survival rate of 96% in an upper-middle-income country (15). The AJCC Ophthalmic Oncology Task Force reported outcomes of patients with diagnosed retinoblastoma in 14 countries (16). The 5-year survival rate was 99% for patients in high-income countries, 89% in upper-middle-income countries, and 90% in lower-middle-income countries. Similarly, the Global Retinoblastoma Study Group analyzed 4,064 children from 149 countries. The 3-year survival rate was 99.5% for children from high-income countries (0.8% had extraocular disease at diagnosis), 91.2% for children from upper-middle-income countries (4.5% had extraocular disease at diagnosis), and 80.3% for children from lower-middle-income. The independent factors identified as indicators of worse survival rates were residence in low-income countries, cT4 advanced tumor, and older age at the time of diagnosis (13).

Previous studies have shown the causes of mortality among Rb patients to be related to metastatic disease (mainly to the CNS or bone marrow), subsequent malignancies (mainly among hereditary Rb survivors who received radiotherapy), and other non-tumor-specific causes, such as infections, endocrine and metabolic diseases, neurological diseases, circulatory diseases, and others (17–21). Even though the mortality rate for Rb in Jordan decreased from 38 to 5% after implementing a telemedicine-based twinning program and strict centralization of care for all Rb patients into a single, specialized tertiary cancer center (3), some patients are still dying from this disease. Herein, we analyzed the causes of death among Rb patients in the setting of advanced centralized care for Rb in a specialized tertiary cancer center [King Hussein Cancer Center (KHCC), Amman, Jordan].

## Methods

The data collected are derived from a retrospective cohort study of 478 Rb patients treated at the King Hussein Cancer Center (KHCC) from 2003 to 2019. All patients had a clinical diagnosis of Rb. Study inclusion required access to patient medical records. The data collected included patient demographics, tumor features and stage at diagnosis, treatment modalities, survival, and causes of death. This study acquired IRB approval from KHCC’s institutional review board (20KHCC08), who also approved waiving the consent form for this retrospective study, and this study complied with the Declaration of Helsinki. Study participants included all patients treated at our service who had a clinical or pathological diagnosis of Rb.

We have included all Rb patients who were followed for at least 3 years after the last active treatment and the patients who passed away from the disease during any follow-up period. Patients on active therapy and patients who were followed for less than 3 years after the last active treatment were excluded from this study. Active treatment was defined as chemotherapy, radiotherapy, and focal consolidation therapy.

### Tumor features, definitions, clinical staging, and treatment modalities

**Figure 1:**
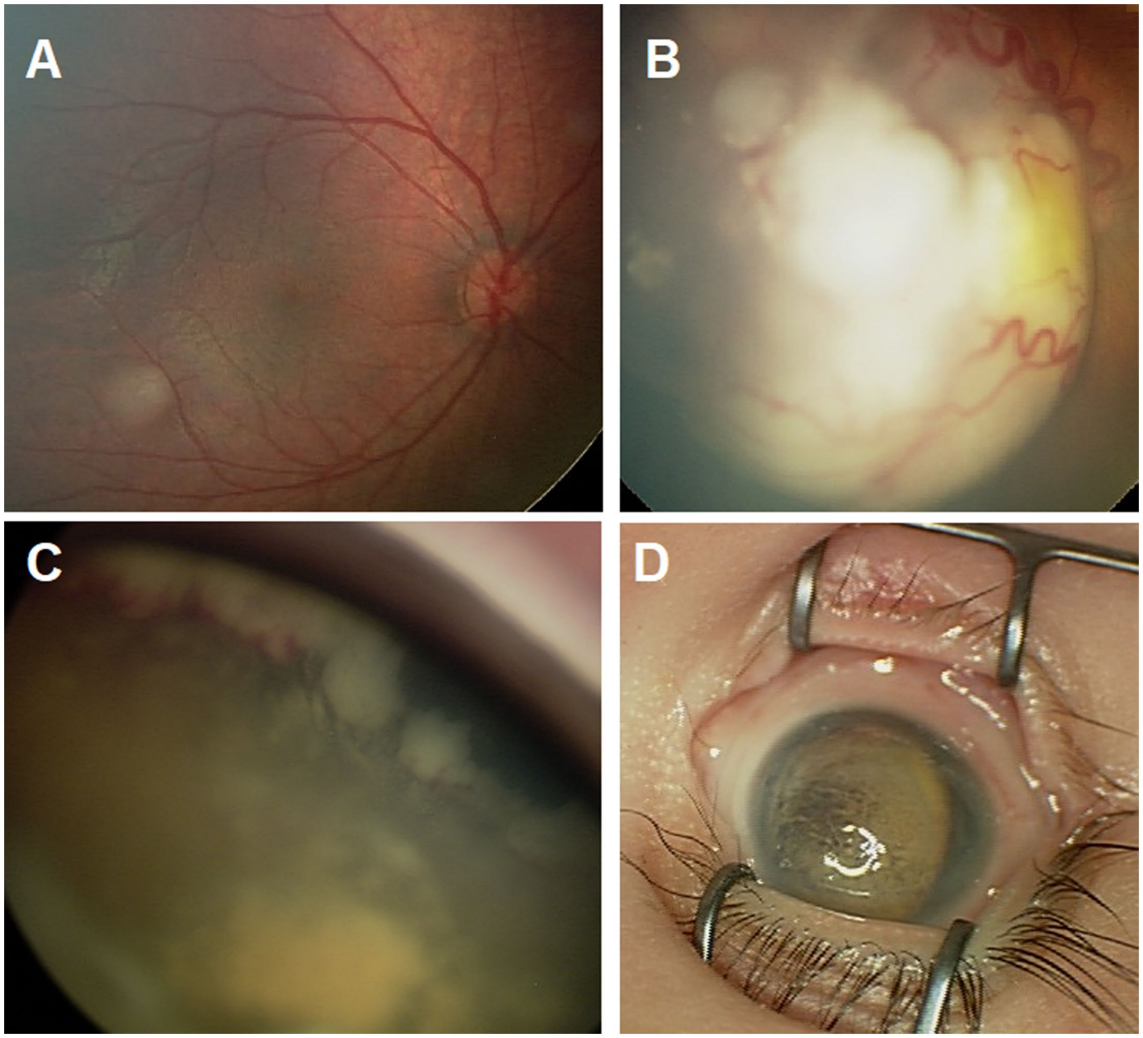
Ret Cam images for the fundus showing group A tumor **(A)**, group C tumor **(B)**, group D tumor with extensive anterior sub retinal seeds **(C)**, and group E eye with phthisis **(D)**.

We reviewed RetCam images and clinical drawings for eyes at the time of diagnosis, documented tumor features, and independently staged the tumors according to both the International Intraocular Retinoblastoma Classification (IIRC) and the eighth edition of the AJCC/UICC cTNM staging systems for Rb (Figure 1) (22, 23). Enucleation was the primary treatment for all group E eyes (T3). The standard eye salvage treatment involved a systemic chemotherapy regimen and focal consolidation therapy. Ocular oncology follow-up was provided, with examination under anesthesia before each cycle of chemotherapy and every 3–4 weeks thereafter. Fundus photos were acquired by using a RetCam II (Clarity Medical System, Pleasanton, CA, United States). Focal therapy was applied when needed as transpupillary thermotherapy. Triple freeze–thaw cryotherapy (MIRA CR 4000) was started after a second cycle of systemic chemotherapy. IAC, IViC, and I^125^ radioactive plaque brachytherapy were used as second-line treatment options in incidences of tumor recurrence or residual tumor activity; both were indicators of primary treatment failure in this study.

### Statistical analysis

Statistical analysis of survival was correlated to demographics and tumor features. The *p* value was measured to test the predictive power of each factor by using Fisher’s exact test; values of 0.05 or less were considered significant. A multivariate logistic regression was performed to assess the relation between mortality and the explanatory variables: Gender, Nationality, Laterality, Family history, IIRC Stage, TNM stage, and presence of high-risk pathology in the enucleated eyes. The multivariate analysis was done using a logistic regression model. A significance criterion of *p* ≤ 0.05 was used in the analysis. All analyses were performed using SAS version 9.4 (SAS Institute Inc., Cary, NC). Kaplan–Meier Survival Analysis was done to calculate the 5-year survival.

## Results

We analyzed clinical data from 478 patients: 249 (52%) were boys, 335 (70%) had bilateral disease, 183 (36%) were Jordanian and 295 (64%) were non-Jordanian. The IIRC stage for the worst eye was A, B, or C for 90 (19%) patients; D for 313 (65%) patients; and E for 73 (15%) patients; only two patients presented with extraocular disease (Table 1). At the last date of follow-up, 25 patients were deceased, 24 (5%) from metastatic disease or second malignancy. One child with prune belly syndrome (and renal failure) died from a noncancer-related systemic issue and was excluded from this analysis.

**Table 1:** Survival data for the 478 patients with retinoblastoma treated at King Hussein Cancer Center (3/2003–12/2019).

Feature		Number	Deaths number (%)	*p* value^e^
Sex	Male	249	11 (4%)	0.53
	Female	229	13 (6%)	
Nationality	Jordanian	183	8 (4.3%)	0.67
	Non-Jordanian	295	16 (5.4%)	
Laterality	Unilateral	147	8 (7%)	0.597
	Bilateral	331	16 (4%)	
Family history	Positive	66	3 (4.5%)	0.518
	Negative	412	21 (5%)	
IIRC^a^ stage of the worst eye at diagnosis	A, B, C	90	1 (1%)	0.0001
	D	313	6 (2%)	
	E	73	15 (21%)	
	Extraocular	2^b^	2 (100%)	
TNM stage of the worst eye at diagnosis	T1	35	0 (0%)	0.0001
	T2	368	7 (2%)	
	T3	73	15 (21%)	
	T4	2^b^	2 (100%)	
Primary treatment	Accepted	474	20 (4%)	0.0001
	Refused	4	4 (100%)	
Primary enucleation (268 eyes)^c^	With HRF^d^	124	14 (11%)	0.013
	Without HRF	144	4 (3%)	

a IIRC, International Intraocular Retinoblastoma Classification.

b Two had extraocular disease diagnosis, and four of the patients who had intraocular disease at diagnosis refused treatment and later on came back with extraocular disease.

c Overall, 268 eyes were treated by enucleation.

d HRF, High-risk pathologic features.

e The *p* value was measured by using Fisher’s exact test.

### Demographics, tumor features, and treatments for the mortality group

For the 24 patients with Rb who died and are included in this analysis, the mean age at diagnosis was 15 months (median, 18 months; range, 4–38 months); 8 (33%) were diagnoses with Rb within the first year of life. There were 11 males (46%) and 13 females (54%). Sixteen (67%) patients had bilateral disease, and three (13%) had a positive family history.

The IIRC stage for the worst eye was A, B, or C for 1 patient (4%); D for 6 patients (25%); and E for 15 patients (63%). Only two patients had extraocular Rb at diagnosis. Zero were T1, 7 (29%) were T2, 15 (63%) were T3, and two (8%) were T4.

One patient’s worst eye was group C at diagnosis; they received conservative therapy and external beam radiation therapy (EBRT), and the eye was not enucleated. Although no metastasis was present, a second malignancy developed. Of the six patients with a group D eye, four received conservative therapy, and consequently, two of these four were salvaged and two were treated by secondary enucleation. Two of the six patients with group D eyes were offered primary enucleation; one patient accepted, but the other declined primary enucleation, returned later with extraocular disease, and then received secondary enucleation. Four of the 6 patients with group D eyes received enucleation (1 primary and 3 secondary). All 15 patients with group E eyes were offered primary enucleation: 12 accepted the offer, and 3 rejected it and returned later with extraocular disease. Of these three patients, two received secondary enucleation, and one died before enucleation. Therefore, 14 eyes in the group E cohort received enucleation (12 primary, and 2 secondary). Notably, two patients had extraocular disease at diagnosis and received enucleation.

The six patients with extraocular disease (2 at presentation, 3 after treatment abandonment in group E patients, and 1 in group D after treatment abandonment) received neoadjuvant chemotherapy. Five were consequently enucleated and received orbital EBRT; one (in group E) died of metastatic disease before undergoing the enucleation or EBRT. A second malignancy developed in one patient in group D and one more in group E (both received EBRT). Pathologically, high-risk pathologic features were seen in 16 of 20 enucleated eyes in 20 patients (Table 2).

**Table 2:** Staging, treatment, and causes of death for 24 Retinoblastoma patients who died in our series.

Characteristics (total 24 patients)		Number (%)
Stage of the worst eye (IIRC)^a^	A,B,C	1 (4%)
	D	6 (25%)
	E	15 (63%)
	Extraocular	2 (8%)
Stage of the worst eye (TNM)	T1	0 (0%)
	T2	7 (29%)
	T3	15 (63%)
	T4	2 (8%)
Treatment modalities	Primary enucleation	13 (59%)
	Primary enucleation (refused)^c^	4 (17%)
	Secondary enucleation	5 (21%)
	Neoadjuvent systemic chemotherapy	14 (60%)
	EBRT^e^	8 (33%)
High-risk features (for 18 enucleated eyes)	No	4 (17%)
	Optic nerve (post laminar)	6 (25%)
	Choroid (massive)	7 (29%)
	Anterior chamber/ ciliary body	2 (8%)
Cause of death	Metastasis^b^	21 (88%)
	Second malignancy^d^	3 (12%)
Site of metastasis (21 patients)	CNS	11 (52%)
	Bone marrow	7 (33%)
	Multi organ metastasis	3 (14%)
Median time between diagnosis and death		34 months
Median time between metastasis and death		6 months

a IIRC, International Intraocular Retinoblastoma Classification.

b One had metastatic pineoblastoma.

c The primary treatment plan for these 4 patients was enucleation but parents refused decision and came back later on with extraocular disease.

d Three patients had second malignancy (1 osteosarcoma in the thigh, 1 osteosarcoma in the orbit, and 1 liposarcoma in the back).

e EBRT, External beam radiation therapy.

**Figure 2:**
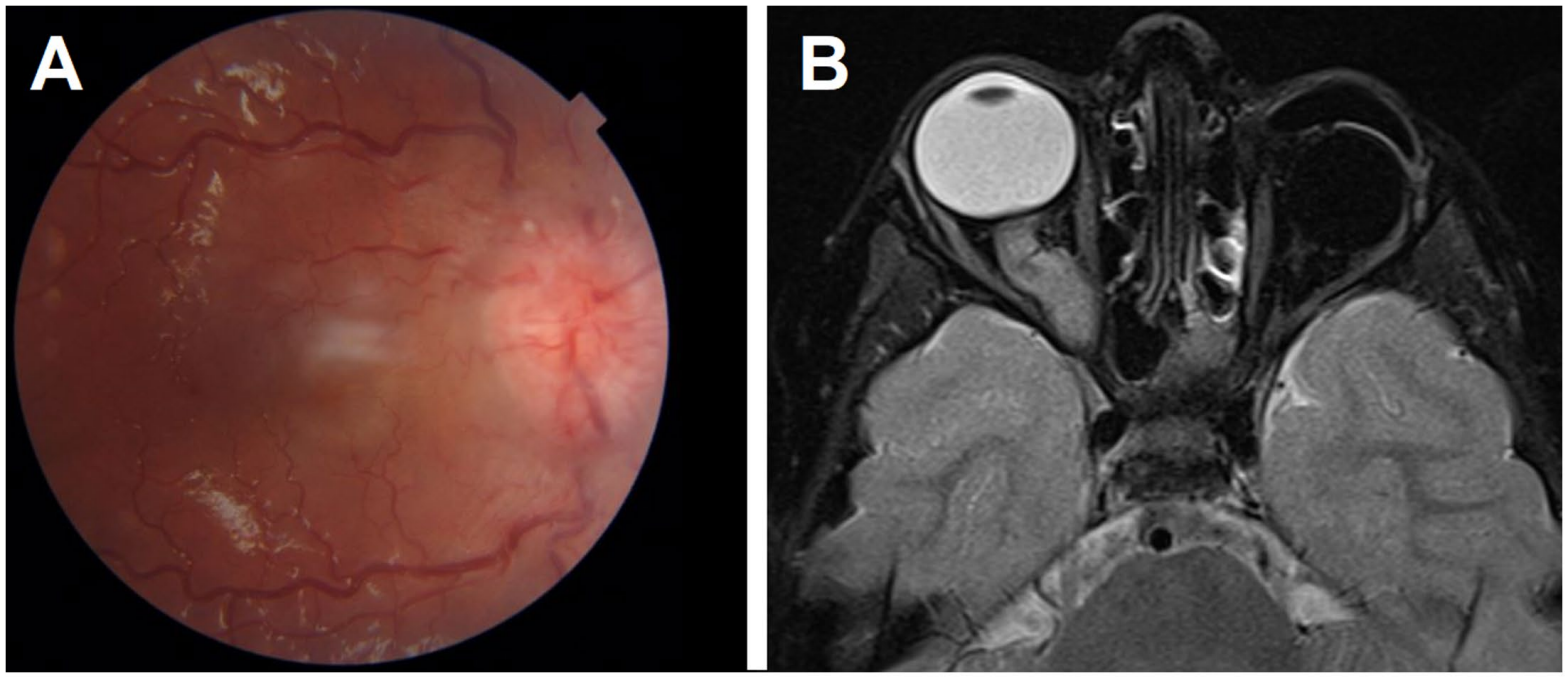
A patient with left unilateral retinoblastoma had her eye enucleated and presented later with right optic disc edema **(A)**. T2 MRI showed thickening and contrast enhancement of the right intra-orbital optic nerve indicating metastatic Retinoblastoma. This was associated with leptomeningeal metastasis **(B)**.

After a 120-month median follow-up period, 24 patients (5%) were dead. Three (5%) had second malignancy (1 osteosarcoma in the thigh, 1 osteosarcoma in the orbit, and 1 liposarcoma in the back), and 21 had metastatic disease. The most common site of metastasis was the CNS (*n* = 11, 52%) (Figure 2), followed by bone marrow (*n* = 7, 33%). One child had CNS metastasis with pineoblastoma. Secondary AML did not develop in any patients.

### Predictive factors of survival

**Figure 3:**
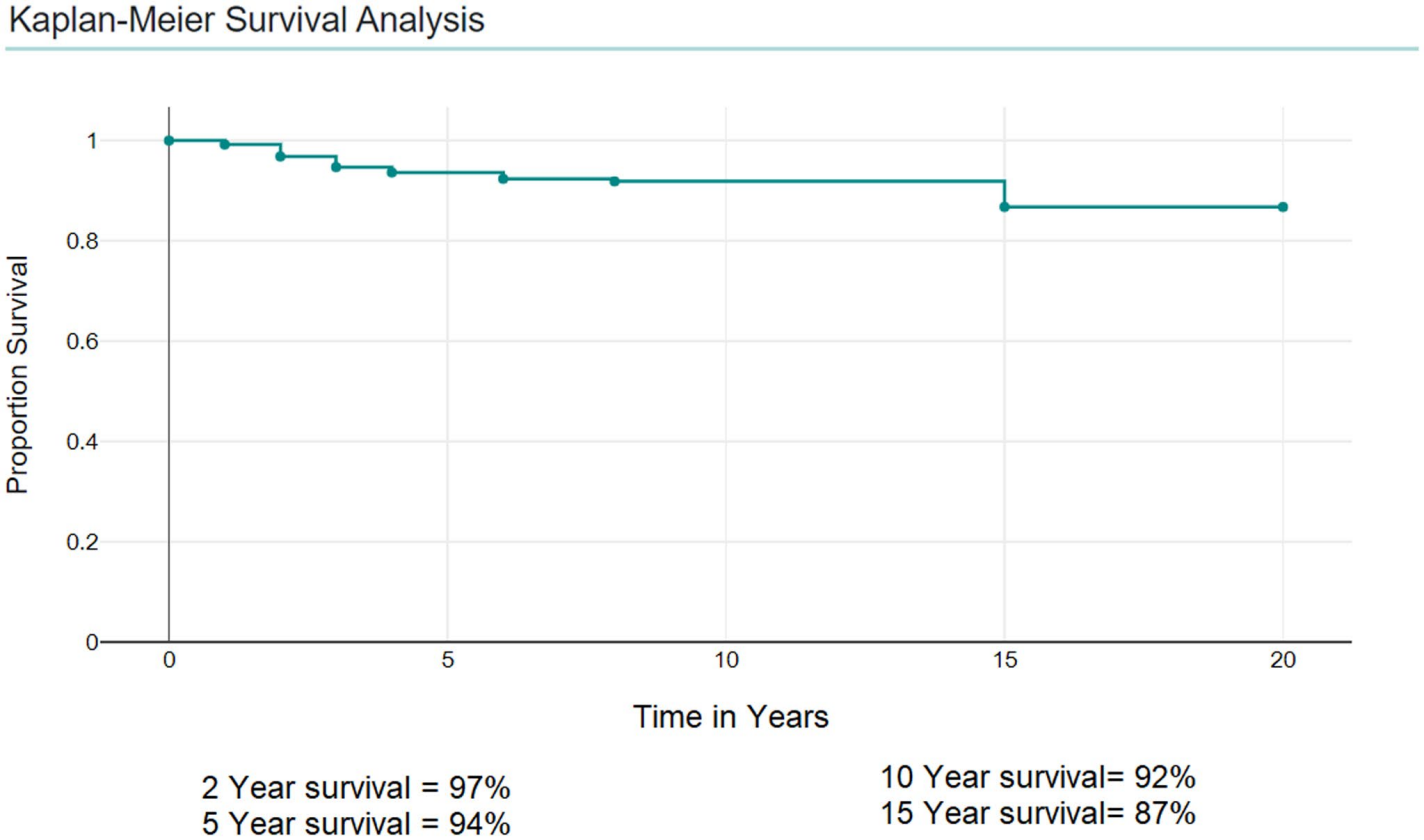
Kaplan–Meier survival analysis for patients with retinoblastoma.

Kaplan–Meier Survival Analysis showed that the 5-year survival was 94%, the 2-year survival was 97%, and the 10-year survival was 92% (Figure 3).

The patient’s sex, tumor site, laterality, nationality, and family history were not significant predictive factors for survival (Table 1). Significant predictive factors for metastasis and death include advanced IIRC tumor stage (*p* < 0.0001), the presence of high-risk pathological features in the enucleated eyes (*p* = 0.013), parental refusal of the specialized team’s recommended primary treatment plan (*p* < 0.0001), and extraocular extension at the time of diagnosis (Table 1). Overall, 30 patients received EBRT; a second malignancy (4 osteosarcomas and 1 liposarcoma) developed in 5 patients, 3 of whom died. All secondary malignancies developed in patients who had received EBRT. Jordanians and non-Jordanians experienced no difference in mortality.

Odds ratio (OR), 95% Wald Confidence Limits, and *p* values were measured in a logistic regression multivariate analysis. Based on that, tumor stage according to the international classification (IIRC) [OR = 31.722, (10.119; 99.450), *p* = 0.0001], tumor TNM stage [OR = 33.445, (10.665; 104.877), *p* 0.0001], and refusal of primary treatment plan [OR = 106.835, (14.935; 764.241), *p* = 0.0001], were associated with higher rates of death.

## Discussion

The estimated mortality rates for retinoblastoma are 60% in low-income, 33% in lower-middle-income, and 21% in upper-middle-income countries (24–26). By contrast, rates in Europe, Canada, and the United States are 3%–5% (4, 26–30). Similarly, the Global Retinoblastoma Study Group recently analyzed 4,064 patients with retinoblastoma from 149 countries and showed a significant disparity in the survival rate of children with Rb depending on the economic level of their country of residence. They found that children with Rb born in low-income countries are 17 times more likely to die from the disease than those born in high-income countries, and only half of the children with Rb in low-income countries remained alive 3 years after diagnosis (13).

One contributing reason driving poor survival outcomes in low-income countries is a delayed diagnosis, with these patients presenting with more-advanced tumors. The American Joint Committee on Cancer Ophthalmic Oncology Task Force analyzed data from a multicenter, international, internet-based registry to determine the risk of metastatic death from advanced intraocular Rb at initial diagnosis. They found that the risk of metastatic mortality correspondently rose with increasing cT subcategory (*p* < 0.001) and that primary enucleation offered the highest survival rates for patients with advanced intraocular Rb (31). Similarly, in our series, no single patient who had the most-advanced tumor in group T1 developed metastasis, but 21% of patients with T3 eyes developed metastasis. All patients who refused primary enucleation from group E eyes died from metastatic disease. In the era of intra-arterial and intra-vitreal chemotherapy, some centers offered eye salvage therapy for group E eyes with more than a 50% salvage rate after extensive therapy (15, 32–34). However, this should be offered with caution to avoid the possible increased risk of metastasis for this group of patients as high-risk histopathologic features predisposing to an increased risk of systemic metastatic disease are present in 18.5%–60% of enucleated group E eyes. Moreover, the visual prognosis for these eyes is extremely poor, and delayed enucleation is associated with lower disease-specific survival (35–40). Therefore, we performed enucleation for all high-risk Rb cases in our series, except for patients with single eyes and some visual acuity.

A second reason behind poor survival outcomes in low-income countries is the limited number of treatment facilities offering radiotherapy and enucleation as the most frequently used modalities. Cancer care and the care of rare diseases may not be top healthcare priorities in many developing countries due to demands for resources and financial support from the agricultural and housing sectors. Limited funding inhibits capacity and support for Rb care in most developing nations in the Middle East and North Africa (39), explaining the expectations for poorer outcomes in developing countries (13). This creates an ethical dilemma, as retinoblastoma is curable if diagnosed early and treated adequately. Jordan is an example of a middle-income developing country where Rb mortality rates decreased to levels similar to those among high-income countries following a build-up in capacity for Rb care in a specialized centralized institute (3). This development indicates that building enough capacity in low- and middle-income countries can improve the outcomes for rare, life-threatening conditions. The benefits of building capacity and centralizing care in low-income countries include increasing awareness of the disease and offering ease of access to health care (3, 36–38). In the low-income countries of Uganda, Senegal, and Nepal, survival rates are 60% (41), 53% (42), and 24% (43), respectively, with metastatic spread reported as the cause of most deaths in all three studies. Survival rates were much higher in developed countries; 99% in the United States (44), 100% in the United Kingdom (45), and 95% in Japan (46); most deaths resulted from trilateral Rb or second malignancies.

Of interest in our series is that even though the mortality was 5%, a rate comparable to high-income countries, metastasis remains the most common cause of death. The contribution of second tumors is low, possibly due to the relatively short follow-up time for survivors. With the centralized expansion of the Rb service, longer post-treatment survivorship clinics are needed to detect patients with second malignancies. Furthermore, there is a tendency to decrease the use of EBRT to a minimum to reduce long-term side effects. All three patients with second malignancies had received EBRT in this series. However, more work is needed to prevent treatment abandonment in low-income countries, as four of 24 patients who died in our series had refused the proposed treatment plan only to return with more advanced and metastatic disease.

We analyzed Rb patients who died from disease-related issues; however, one patient in our cohort had prune belly syndrome and died of renal failure, which was not related to his cancer (47). Similar analyses of cause-specific mortality in RB patients that were not due to tumors have uncovered leucopenia (48), septicemia (49), and circulatory or cardiovascular disease (50). Neutropenia, transient fever, and nausea/vomiting are the most common systemic complications of IVC and IAC, whereas retinal detachment is the most common ocular complication (51–54). Treating physicians should consider these factors as possible causes of death other than metastasis during the treatment of patients with Rb (55).

In our practice, primary enucleation is more likely to be offered to patients with unilateral Rb than to those with bilateral Rb (3); therefore, we may expect more-delayed enucleation and increased chance of high-risk pathologic features and then increased risk of metastasis for patients with bilateral disease than for those with unilateral disease (56–58). However, the mortality was 7% for unilateral Rb and 4% for those with bilateral disease, a difference that is not statistically significant (*p* = 0.59). Treatment rejection might be a culprit: three of the four patients who refused primary enucleation in our series had unilateral disease, explaining the non-significant difference. The American Joint Committee on Cancer Ophthalmic Oncology Task Force reported similar findings of primary enucleation more commonly selected as treatment in unilateral, advanced Rb than in cases of bilateral disease (31, 36). This strategy may minimize the metastatic risk in unilateral patients; however, the treatment modality and tumor laterality analysis did not show a significant difference between metastasis-related deaths of those with unilateral tumors and those with bilateral tumors in the same treatment arm. We found that advanced age at presentation was a poor prognostic factor for survival. This finding is supported by evidence from a study establishing a correlation between increasing age with high-risk genomic features (59).

The centralization of care for all Rb patients in a single center in a small developing country (Jordan) enhanced earlier diagnosis, reduced mortality, improved eye salvage rates, and improved screening for at-risk children. This is because the patients now have straightforward, timely access to adequate healthcare that improved their long-term outcomes. The average at diagnosis for Rb in Jordan dropped from 13 months to 6 months for bilateral cases and from 32 months to 28 months for unilateral cases (3, 60–65). In conclusion; the 5-year survival rates of Rb patients in Jordan are as high as those in high-income countries. In this study, we have shown that even with limited resources, managing rare diseases exclusively at specialized referral centers with adequate capacity can offer Rb patients outcomes that are similar to those in high-income countries. Rb patients are, however, still dying from metastatic disease, prompting the need for awareness campaigns to educate the public about the high cure rates, and preventing treatment abandonment. Our study has limitations that must be considered. First, this was a retrospective study with a limited number of patients. Next, all patients are from a single institution; thus, the findings might not be generalizable to the entire region. However, this typical weakness could also be considered a strength in this case because the institution exclusively treats all Rb patients in the country. Most importantly, they have changed the natural history of Rb in the country, taking it from a lethal to a curable disease.

## Data availability statement

The raw data supporting the conclusions of this article will be made available by the authors, without undue reservation.

## Ethics statement

The studies involving humans were approved by King Hussein Cancer Center IRB. The studies were conducted in accordance with the local legislation and institutional requirements. The ethics committee/institutional review board waived the requirement of written informed consent for participation from the participants or the participants’ legal guardians/next of kin because retrospective study for patients who are dead.

## Author contributions

YY, TB, RA, MO, and MuM: conceptualization, formal analysis, and data curation. TB, YY, IJ, HH, AA, DA, and NA: methodology and investigation. YY, MoM, and IA-N: validation, resources, writing—review and editing, and supervision. YY, TB, RA, MO, MuM, IJ, HH, AA, DA, and NA: writing—original draft preparation. All authors contributed to the article and approved the submitted version.

## Funding

This research was supported in part by King Hussein Cancer Center, Amman, Jordan.

## Conflict of interest

The authors declare that the research was conducted in the absence of any commercial or financial relationships that could be construed as a potential conflict of interest.

## Publisher’s note

All claims expressed in this article are solely those of the authors and do not necessarily represent those of their affiliated organizations, or those of the publisher, the editors and the reviewers. Any product that may be evaluated in this article, or claim that may be made by its manufacturer, is not guaranteed or endorsed by the publisher.

## Abbreviations

AJCC: American Joint Committee on Cancer
EBRT: External Beam Radiation Therapy
IAC: Intra Arterial Chemotherapy
IIRC: International Intraocular Retinoblastoma Classification
KHCC: King Hussein Cancer Center
OR: Odds Ratio
Rb: Retinoblastoma
RB1: Retinoblastoma 1 Gene
